# Supplementary figures and images for: Molecular determinants of Neu5Ac binding to a tripartite ATP independent periplasmic (TRAP) transporter
Source: eLife. 2025 Feb 6;13:RP98158. doi: 10.7554/eLife.98158 (PMC11801797; doi:10.7554/eLife.98158)

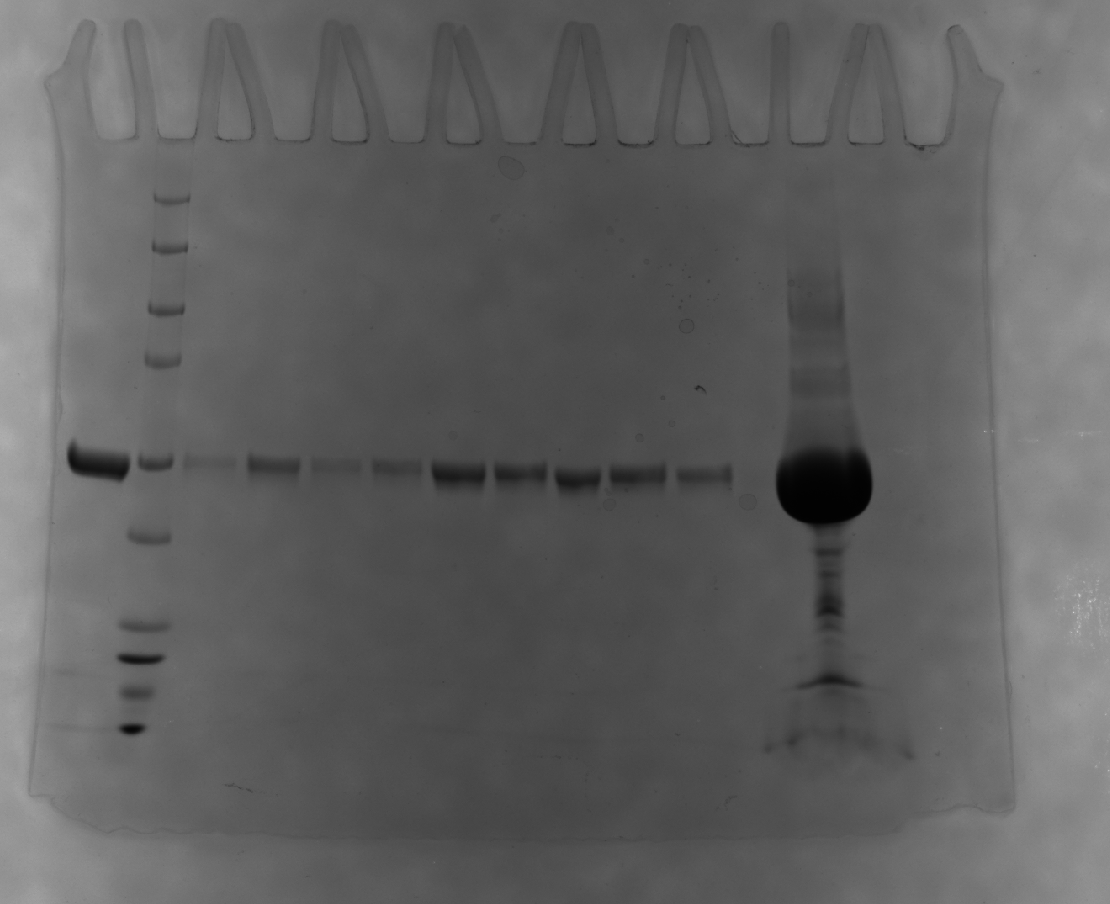

Supplement: Figure 1—figure supplement 1—source data 1. [file elife-98158-fig1-figsupp1-data1.tif]

Figure 1—figure supplement 1 -inset

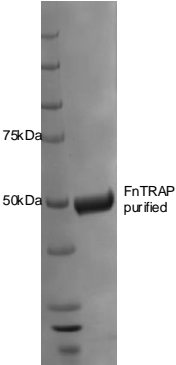

Raw Gel:

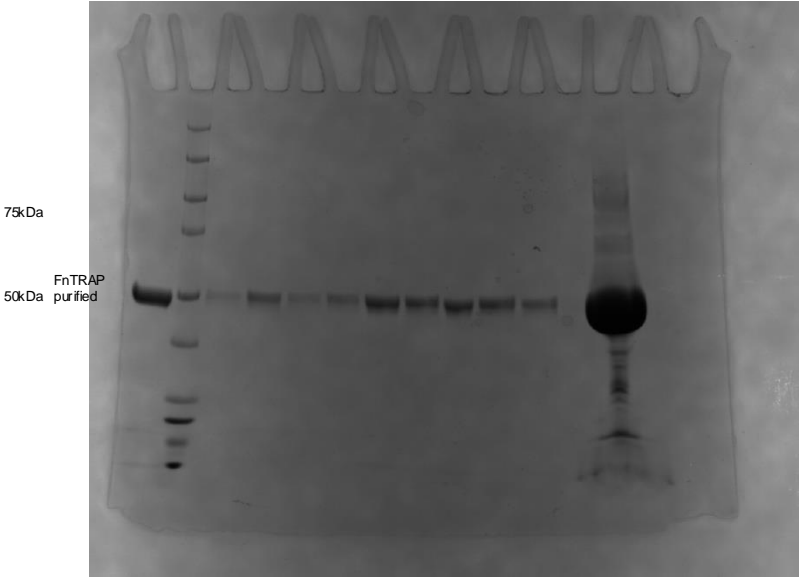

Flip 180  
degrees  
→

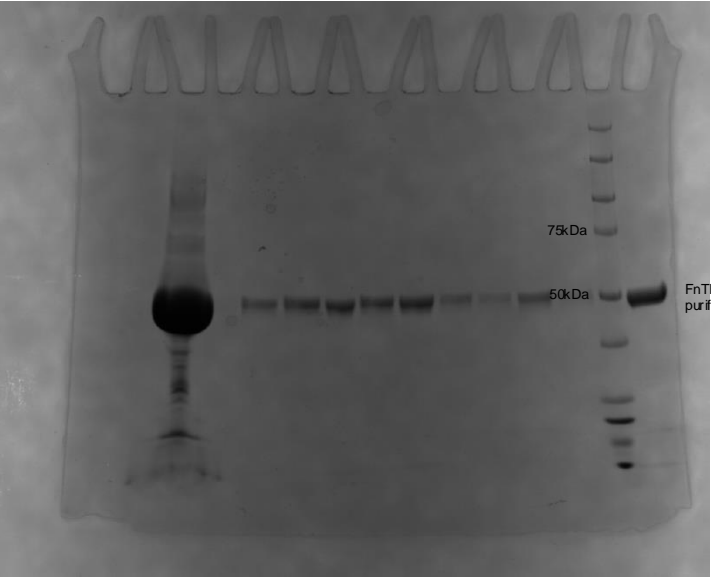

Crop  
→

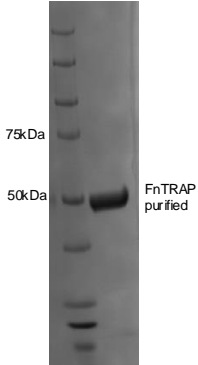

Supplement: Figure 1—figure supplement 1—source data 2. [file elife-98158-fig1-figsupp1-data2.pdf]
